# Rapid nanocatalytic approach for azo dye degradation using bi-ligand nickle based-metal organic frameworks

**DOI:** 10.1186/s13065-025-01650-8

**Published:** 2025-10-27

**Authors:** Aya A. Mouhamed, Ahmed Elsayed, Noha Mostafa, Amr M. Mahmoud, Amr Elshaer, Aya T. Soudi

**Affiliations:** 1 Pharmaceutical Analytical Chemistry Department, Faculty of Pharmacy, Cairo University, El-Kasr El-Aini Street, Cairo, 11562 Egypt; 2 Department of Chemistry, School of Pharmacy, Newgiza University, Giza, Egypt; 3 Drug Discovery, Delivery and Patient Care (DDDPC), School of Life Sciences, Pharmacy and Chemistry, Kingston University London, Kingston Upon Thames, Surrey, KT1 2EE UK

**Keywords:** Azo-dyes, Bi-ligand Ni-MOFs, Environmental remediation, Metal organic frameworks, Methyl orange

## Abstract

A novel bi-ligand nickel-based metal–organic framework (Ni-BTC-PYDC MOF) was synthesized using benzene tricarboxylic acid (BTC) and pyridine-2,3-dicarboxylic acid (PYDC) as ligands. This MOF showed improved surface area, structural stability, and electron transfer compared to mono-ligand Ni-MOFs. Characterization by SEM, EDX, EDS mapping, XRD, and FT-IR confirmed its enhanced morphology and nickel content. The catalyst rapidly reduced methyl orange (MO) dye in water, achieving rapid and significant decolorization within 90 s using sodium borohydride (NaBH₄) under mild conditions. It maintained high activity over ten reuse cycles with minimal loss, performing best at pH 5 due to efficient hydride generation and proton-assisted electron transfer. These findings demonstrate that the bi-ligand Ni-MOF is a promising, stable, and reusable catalyst for removing toxic azo dyes from wastewater.

**Supplementary Information:**

The online version contains supplementary material available at 10.1186/s13065-025-01650-8.

## Introduction

Azo dyes are synthetic organic compounds containing an azo group (–N = N–) linking aromatic rings. They account for nearly 70% of global dye production and are widely used in textiles, leather, paper, and cosmetics for their stable, vibrant colors. However, they pose serious environmental and health risks, being carcinogenic, mutagenic, and resistant to biodegradation, which leads to persistent water pollution. When released into water bodies, their intense color blocks sunlight, hindering photosynthesis in aquatic plants and disrupting ecosystems, often reducing oxygen levels and biodiversity. Untreated wastewater containing azo dyes can also contaminate drinking water sources, making their degradation a priority in environmental research [1, 2]. Tackling these problems, the breakdown of azo dyes has emerged as a focus in environmental science research.

Over the years, various physical, chemical, and biological methods have been employed for azo dye degradation, including adsorption [3–5], photocatalysis [6–9], biodegradation [4, 10, 11], and oxidation processes [12–14]. However, these reported traditional techniques have several drawbacks, such as incomplete degradation, high operational costs, low efficiency, and the generation of secondary pollutants. Consequently, there is a growing demand for more effective and sustainable catalytic systems that effectively degrade azo dyes resulting in reduced environmental pollution. Monitoring and quantifying such degradation is equally important, and recent advances in analytical techniques, such as spectrophotometric approaches [15–17], chromatographic [18–21] and electrochemical sensing strategies [22–26], have demonstrated significant potential in improving detection precision.

Catalytic reduction is a highly effective and eco-friendly method for degrading azo dyes by breaking the azo bond (–N = N–) into smaller, less toxic molecules, resulting in significant decolorization of the dye solution. Sodium borohydride (NaBH₄), a strong electron donor, is often used to reduce azo dyes to non-toxic aromatic amines. For methyl orange (MO), the process is significantly faster and more efficient when a catalyst is combined with NaBH₄, as NaBH₄ alone works slowly and requires higher concentrations and longer reaction times. Catalysts enhance electron transfer, thereby boosting reaction efficiency.

The choice of catalyst plays a crucial role in catalytic reduction reactions. Conventional catalysts often exhibit limitations such as low catalytic activity, poor stability, and high material costs [27]. So, nanocatalysts have emerged as promising materials due to their exceptional properties, including high surface area, enhanced reactivity, and recyclability [28]. These nanoscale catalysts significantly accelerate the reduction process by providing higher surface area and hence more active sites for the reaction, thereby improving the degradation efficiency of azo dyes, which offers superior performance, faster reaction kinetics, and potential reusability. Various nanocatalysts have been reported in the literature for the catalytic reduction of methyl orange (MO), including Cu NPs [29], Pd NPs [30], Ag NPs [31, 32], SDS/Ag NPs [33], CuAg nanoparticles on highly porous ZnO/carbon black-cellulose acetate sheet [34], polyaniline/nickel oxide composite [35], MFe_2_O_4_/γ-Fe_2_O_3_ nanocomposites (Metal = Ni or Co) [36], Fe nanoparticles encapsulated in MOF-derived carbon [37] and Prussian blue analogue [2]. While these catalysts showed good catalytic performance, there is still a need for a faster, stable, reusable, and cost-effective approach. To address this, we explored the use of Ni-BTC-PYDC MOF, a bi-ligand metal-organic framework, as a promising alternative for the efficient catalytic reduction of MO.

Metal-organic frameworks (MOFs) are crystalline porous materials made up of metal ions linked together by organic molecules, creating structures that can be two or three-dimensional. They have gained significant attention due to their unique combination of flexible organic parts and strong metal pieces. Another key feature of MOFs is their porosity and high surface area, making them useful for a variety of purposes [38–41]. They are also very easily tunable, and researchers can make them with desired properties by choosing a range of different metals and organic ligands [42]. They also have a very good chemical and thermal stability, making them usable in various environments [38].

MOFs are widely used for gas storage and separation, enabling the capture and holding of gases [43]. They also serve as effective materials in catalysis, including photocatalysis, asymmetric catalysis, and biocatalysis [44]. Their tunable pore sizes and high surface area provide sufficient sites for reactions to take place. Furthermore, MOFs are employed in drug release systems because they are biocompatible and porous in nature for controlled delivery of drugs [45]. You can also find them in chemical sensors that detect various substances [46], as well as in fuel cells and batteries for the support of energy storage and conversion [47, 48]. MOFs are also applied in detoxification of the environment and aiding in the sustainability of industrial procedures [2]. With all these qualities, MOFs are leading the way in materials research and are advantageous as nanocatalysts when it comes to reducing toxic azo dye pollutants.

Here, we will address the catalytic potential of MOFs with particular focus on bi-ligand metal-based MOFs. Bi-ligand metal-based MOFs are prepared by connecting two different organic ligands and a metal ion in such a way that complex structures are generated that offer greater structural diversity, stability, and catalytic ability [49]. The choice of the ligands and metals plays an important role in deciding the properties of these materials. You can adjust the pore size and other characteristics by changing the proportion of the ligands [50]. Moreover, the interaction among the various ligands in the MOF framework leads to synergistic effects that increase the overall catalytic activity compared to single-ligand MOFs. These interactions lead to increased stability and reactivity, making bi-ligand MOFs a more effective choice for most applications [49, 50].

In this study, we focus on a nickel-based framework prepared with two ligands: benzene tricarboxylic acid (BTC) and pyridine-2,3-dicarboxylic acid (PYDC). This combination of ligands makes the structure more stable and improves its performance. Nickel (Ni) ions contribute to the MOF’s catalytic properties. Since nickel is abundant and cheap, nickel-based MOFs are a budget-friendly option compared to other metal frameworks. So, the bi-ligand Ni-based MOF (Ni-BTC-PYDC MOF) seems like a good candidate as a nanocatalyst in the catalytic reduction of environmental pollutants such as MO azo dye.

## Experimental

### Materials and reagents

The materials used in this study are included in Table S1. The Britton-Robinson buffer (BRB) was prepared on-site, and its pH was adjusted to the required value using a 0.2 M NaOH solution [51].

### Apparatus and software

A Shimadzu UV-1900 double-beam spectrophotometer (Kyoto, Japan) was utilized for obtaining spectroscopic data using precisely matched 1.00 cm quartz cells for the experimental procedure. Spectral measurements were performed over a wavelength range of 300.0 to 600.0 nm, with intervals of 0.1 nm. Identical quartz cuvettes, each with a 1 cm pathlength, were used during the analysis. Data acquisition was automated using the Shimadzu UV-Probe 2.43 software system. The surface morphology of the synthesized materials was analyzed using a scanning electron microscope (SEM, model S-4700, Hitachi, Japan) and High Resolution Transmission Electron Microscope (HRTEM), JEM 2100 HRT, Japan). Powder X-ray diffraction (PXRD) patterns were measured on PANanalytical X-Ray Diffraction equipment model X׳Pert PRO with Secondary Monochromator, Cu-radiation (λ=1.542Å) at 45 K.V., 35 M.A. and scanning speed 0.04o/sec. were used. The diffraction peaks between 2θ = 2o and 60o, corresponding spacing (d, Å) and relative intensities (I/Io) were obtained. The surface area and pore size of the synthesized Ni-MOFs were calculated from N_2_ gas adsorption/desorption isotherm by BET and BJH analysis at 77 K ( BEL sorp-max machine, BEL, Japan). To identify the functional groups present, Fourier transform infrared spectroscopy (FT-IR, Tensor 27, Bruker, Germany) was employed. Furthermore, the elemental composition of the material surfaces was examined through energy-dispersive X-ray spectroscopy (EDX, Japan).

### Synthesis of mono- and bi-ligand Ni-MOFs

**Fig. 1:**
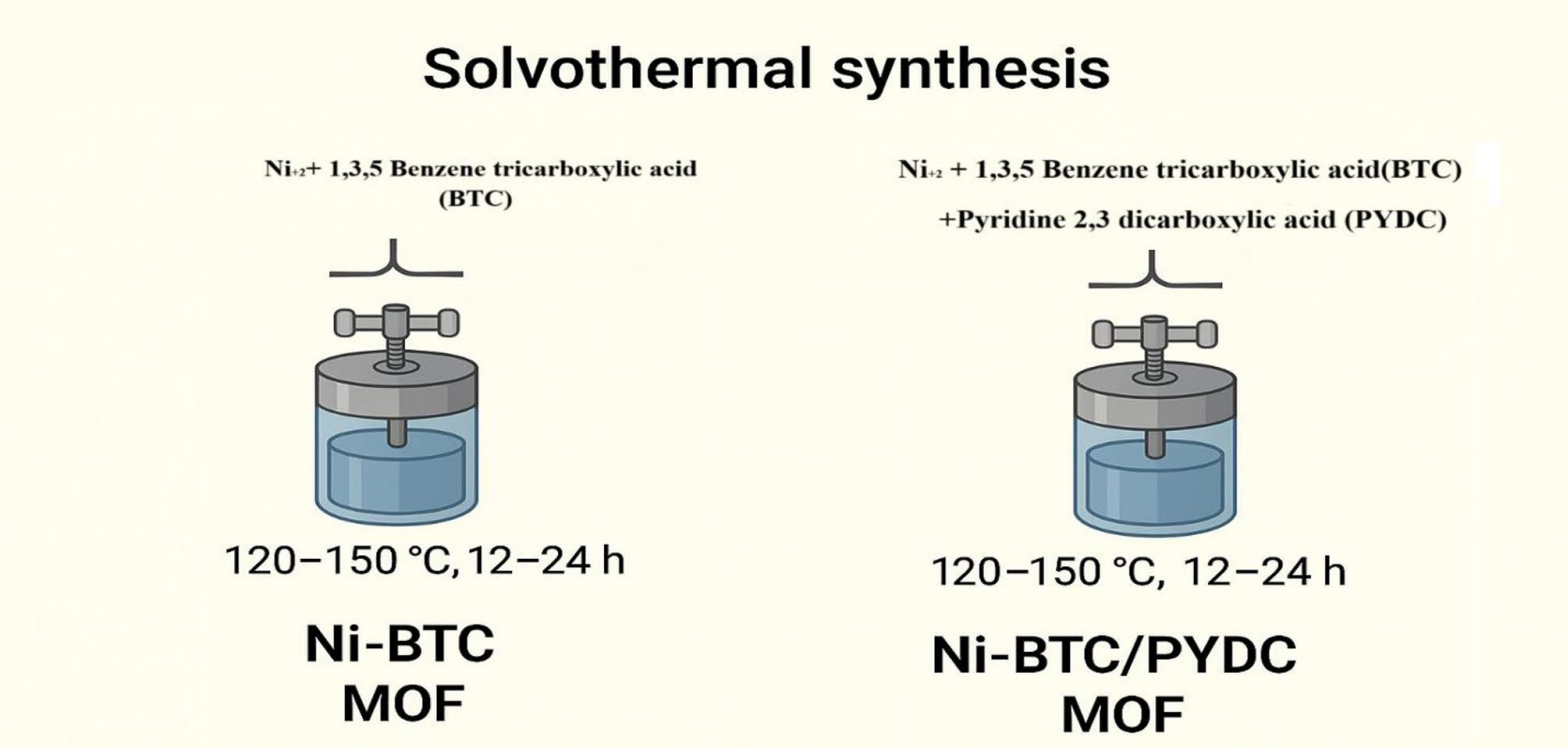
Schematic diagram of the mono-ligand and bi-ligand Ni-MOFs synthesis procedure

The preparation of mono-ligand Ni-MOFs was performed using the solvothermal method, based on a procedure reported in the literature [52], with some modifications. To synthesize the mono-ligand Ni-MOFs, 5.0 mmol of NiCl₂·6 H₂O was dissolved in 5 mL of deionized water. In another container, 5.0 mmol of BTC was dissolved in 30 mL of ethanol. After that, the two solutions were mixed and transferred to a 50-mL Teflon-lined autoclave. The mixture was heated at 180 °C for 12 h. Then, it was allowed to cool naturally to room temperature. The produced solid was collected via centrifugation, washed three times with deionized water, and once with ethanol. The product was dried in an oven before use. For the bi-ligand Ni-MOFs, we followed the same steps but used 2.5 mmol of BTC and 2.5 mmol of PYDC instead of only BTC. The final product obtained was our bi-ligand Ni-MOFs. The percentage yield were calculated based on the mass of the dried product relative to the total mass of reactants used, resulting in yields of 35% for the bi-ligand and 33% for the mono-ligand. A schematic representation of the synthesis process is presented in Fig. 1.

### Investigation of the catalytic performance of mono- or bi-ligand Ni-MOFs in the reduction of MO

The catalytic reduction of the azo dye MO was investigated in an aqueous medium, using NaBH₄ as the reducing agent along with Ni-MOFs as nanocatalysts. UV-vis spectrophotometry was used to measure changes in the MO absorbance peak at 465 nm over time. The experiment was carried out as follows: 0.1 mL of MO solution (3.0 mM) was added into a test tube, followed by 0.2 mL of an aqueous dispersion of either mono-ligand or bi-ligand Ni-MOFs (1 mg/mL), and 0.2 mL of NaBH₄ solution (300.0 mM). UV-vis spectrophotometry was used to monitor the reaction to see how the MO’s absorption peak at 465 nm changed with time. Control experiments were conducted using the same procedure, but without the addition of the Ni-MOFs, to see how they performed in comparison.

### Investigation of Ni-MOFs reusability in the catalytic reduction of MO

To evaluate the applicability of sustainable azo dye treatment, recycling experiments were performed to confirm whether the prepared Ni-MOFs can be reused. Bi-ligand Ni-MOFs’ catalytic activity and stability were examined over multiple cycles, as it’s important for them to keep their shape and performance when used in real life. We repeated the reduction process ten times with the same catalyst each time, just adding a new batch of the starting material after each round without needing to regenerate the catalyst. All experiments were performed in triplicate to ensure reliability. The performance of the Ni-MOFs was tested to know how durable and effective they are on prolonged use.

## Results and discussion

In our study, the catalytic activities of both mono-ligand and bi-ligand Ni-MOFs were investigated for their effectiveness in the reduction of methyl orange (MO) using NaBH₄ as the reducing agent. The hypothesis that bi-ligand Ni-MOFs would exhibit enhanced catalytic efficiency compared to their mono-ligand counterparts was validated through quantitative analysis using UV-Vis spectrophotometry, providing precise measurements of MO degradation.

### Characterization of the synthesized mono- and bi- ligand Ni-MOFs

**Fig. 2:**
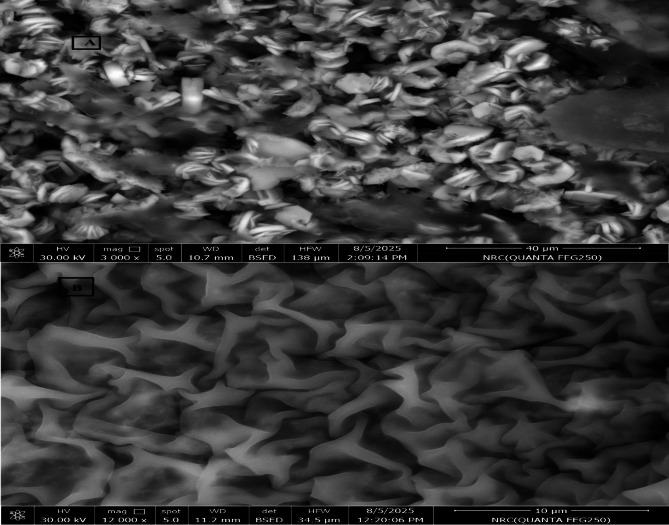
**A** SEM image of bi-ligand Ni-MOFs and ** B** SEM image of mono-ligand Ni-MOFs

The SEM analysis revealed distinct morphological differences between the bi- and mono-ligand Ni-MOFs as shown in Fig. 2. In Fig. 2a, the bi-ligand Ni-MOFs exhibited a uniform spherical morphology with particles ranging approximately from 100 to 200 nm in size. The uniformity also helps in maintaining consistent catalytic performance over a number of cycles because non-uniform particles would lead to variability in performance. The small particle size of these Ni-MOFs offers a larger surface area, where there is better interaction with the MO molecules as well as facilitating higher reaction rates. The particle surface is also even with minimal irregularities that enhance access of active sites, facilitating better contact with reactants during the catalytic reduction process. In contrast, as seen in Fig. 2b, the mono-ligand Ni-MOFs featured particles that were irregular in morphology and had much larger particle size compared to the bi-ligand Ni-MOFs. These bigger, uneven particles have a smaller surface area, which makes it harder for them to interact with MO molecules. Their irregular shape also hinders the ease of access to active sites, thus leading to lower catalytic activity.

To complement the SEM observations, high-resolution transmission electron microscopy (HRTEM) was performed to investigate the internal structure and nanoscale features of both materials. While SEM provided an overview of surface morphology and size distribution, HRTEM offered detailed insight into crystallinity and particle boundaries (Figure S1). The bi-ligand Ni-MOF (Figure S1a) displays well-defined crystalline domains with particle sizes ranging from ~ 36 nm to ~ 69 nm. The uniformity and clear lattice contrast indicate good crystallinity and controlled growth, likely facilitated by the dual-ligand coordination. In comparison, the mono-ligand Ni-MOF (Figure S1b) exhibits a broader size distribution, with features from ~ 11 nm to over 116 nm. The presence of larger aggregates alongside smaller particles suggests less uniform nucleation, which may contribute to variations in surface area and active site accessibility. These observations support the conclusion that incorporating both BTC and PYDC ligands promotes more homogeneous particle formation and better structural ordering than the single-ligand system.

**Fig. 3:**
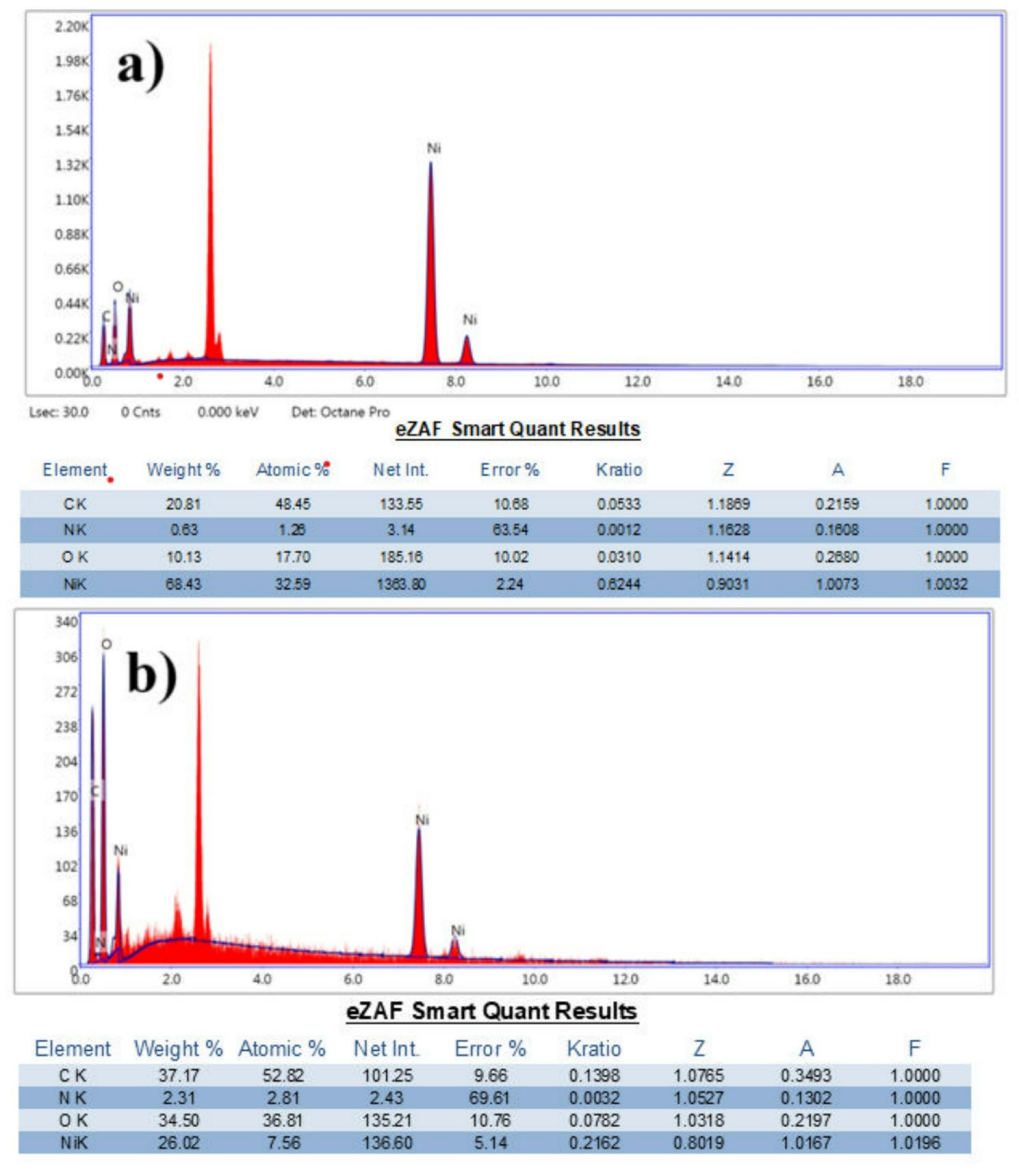
**a** EDX of bi-ligand Ni-MOFs, and **b** EDX of mono-ligand Ni-MOFs

The elemental composition of both mono-ligand and bi-ligand Ni-MOFs was investigated through EDX analysis. It showed that nickel (Ni), carbon (C), and oxygen (O) are present in both types (Fig. 3). These elements are key parts of the MOF structure, with nickel acting as the metal core and carbon and oxygen coming from the organic ligands. We noticed a big difference in the nickel content between the two materials. The bi-ligand Ni-MOFs (Fig. 3a) had a much higher nickel content than the mono-ligand ones. This difference highlights the superior binding efficiency of the ligands in the bi-ligand system, which translates into a more robust and active catalyst. The reduced nickel content in the mono-ligand Ni-MOFs (Fig. 3b) not only affects the density of active sites but also compromises the material’s overall performance and stability. Moreover, to verify the elemental distribution within the MOF structures, EDS mapping was performed for both the mono-ligand and bi-ligand materials (Figure S1&S2). The elemental maps show that Ni, C, O, and N are uniformly distributed across the crystal surfaces. In the bi-ligand Ni-MOFs (Figure S2), nickel appears evenly dispersed without noticeable aggregation, while the faint nitrogen signal confirms the successful incorporation of the PYDC ligand. The mono-ligand Ni-MOFs (Figure S3) also exhibit homogeneous elemental dispersion; however, they contain a greater proportion of carbon and oxygen relative to nickel. These observations, in agreement with the bulk composition analysis, indicate that the use of a second ligand increases Ni content and promotes a more uniform distribution of active sites compared to the mono-ligand framework.

**Fig. 4:**
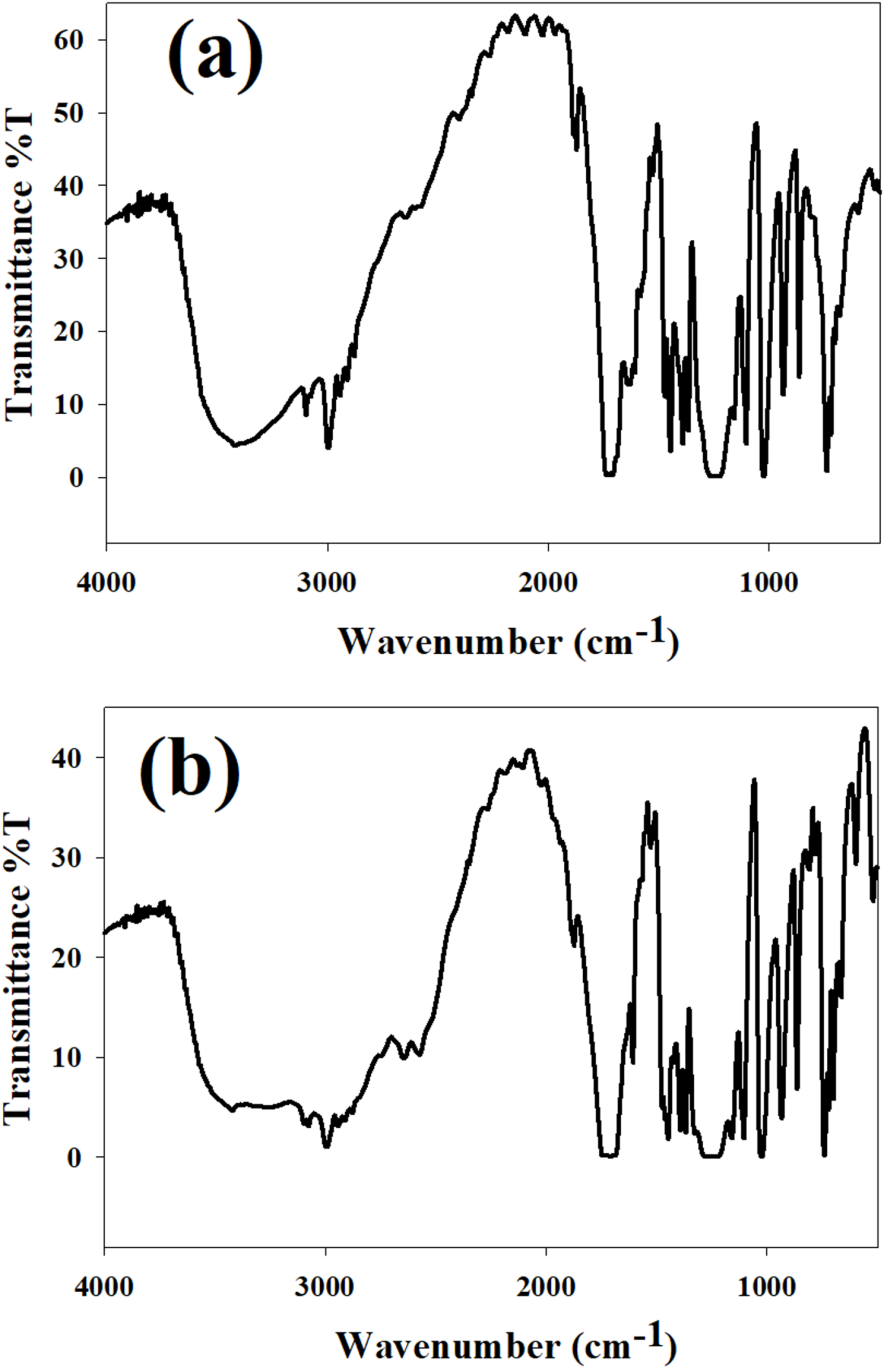
**a** FT-IR spectra of bi-ligand Ni-MOFs, and **b** FT-IR spectra of mono-ligand Ni-MOFs

The electronic structure and coordination sites of both mono- and bi-ligand Ni-MOFs employing FT-IR spectra (Fig. 4) reveal characteristic vibrational modes associated with the organic ligands and metal-ligand interactions, confirming the successful incorporation of carboxylate groups into the MOF frameworks. Key absorption bands were observed at ~ 3400 cm⁻¹ (O-H stretching), ~ 1650 cm⁻¹ (C = O stretch of carboxylate), and 1400–1600 cm⁻¹ (symmetric and asymmetric COO⁻ stretching), along with lattice vibrations below 1000 cm⁻¹ indicative of metal-ligand coordination. Notably, the bi-ligand Ni-MOFs (Fig. 4a) exhibited sharper and more intense peaks compared to the mono-ligand Ni-MOFs (Fig. 4b), suggesting a more ordered and stable framework with enhanced ligand-metal coordination efficiency. Also, the bi-ligand system displayed additional peaks around 1700–1800 cm⁻¹, attributed to secondary interactions or overlapping vibrational modes arising from the presence of multiple ligands. The differences in structure are clear when you look at the broader and less pointed peaks in the mono-ligand Ni-MOFs. This means that the coordination between the metal and ligands is weaker, leading to a less compact structure.

The textural properties of the mono- and bi-ligand Ni-MOFs were investigated through nitrogen adsorption–desorption isotherms and pore size distribution analyses (Figures S4 & S5). The isotherms of both samples exhibit a sharp increase in nitrogen uptake at high relative pressures (p/p₀ >0.9), characteristic of type IV isotherms with H3-type hysteresis loops, suggesting the presence of mesoporous structures. It should be noted that this steep uptake primarily reflects mesoporosity arising from inter-particle voids, while the intrinsic micropores of the framework contribute to the overall surface area and adsorption capacity. For the bi-ligand Ni-MOFs (Figure S4a), the Brunauer–Emmett–Teller (BET) surface area was determined to be significantly higher, accompanied by a steep adsorption step, indicating extensive mesoporosity in addition to the intrinsic microporous network. The corresponding NLDFT-derived pore size distribution (Figure S4b) revealed a narrow peak centered at approximately 25–26 nm, confirming the uniform mesopore formation. This enhanced surface area and large pore size may be attributed to the synergistic coordination environment created by the simultaneous incorporation of BTC and PYDC ligands, which can promote a more open framework. In contrast, the mono-ligand Ni-MOFs (Figure S5a) exhibited a markedly lower nitrogen uptake and BET surface area, indicating a more compact structure with reduced accessible porosity. The pore size distribution (Figure S5b) showed a sharp peak around 14–15 nm, reflecting smaller mesopores compared to the bi-ligand counterpart. The reduced pore diameter and surface area in mono-ligand MOFs can be attributed to the absence of the secondary PYDC ligand, which limits the expansion of the framework and decreases interstitial void space.

The crystallographic structures of the bi-ligand and mono-ligand Ni-MOFs were examined using powder X-ray diffraction (XRD) as presented in Figure S6. Both samples show sharp diffraction peaks, confirming their crystalline nature. The bi-ligand Ni-MOF (Figure S6a) exhibits intense reflections, indicating a well-ordered framework. The higher peak intensity and sharpness suggest enhanced long-range ordering, likely due to the cooperative coordination of both ligands with Ni centers. In contrast, the mono-ligand Ni-MOF (Figure S6b) retains the main structural features but shows broader, less intense peaks, implying smaller crystallites or partial structural disorder. These results confirm the successful formation of the Ni-MOF phase in both systems, with the bi-ligand material exhibiting superior crystallinity, which can favor stability and catalytic performance.

These findings show that the bi-ligand Ni-MOFs are better when it comes to coordination efficiency and stability, which is important in determining how efficiently they carry out catalytic activities. Mono-ligand Ni-MOFs, on the other hand, possess a simpler ligand structure and are less efficient in coordination, limiting their performance and durability.

### Catalytic reduction experiments of MO

**Fig. 5:**
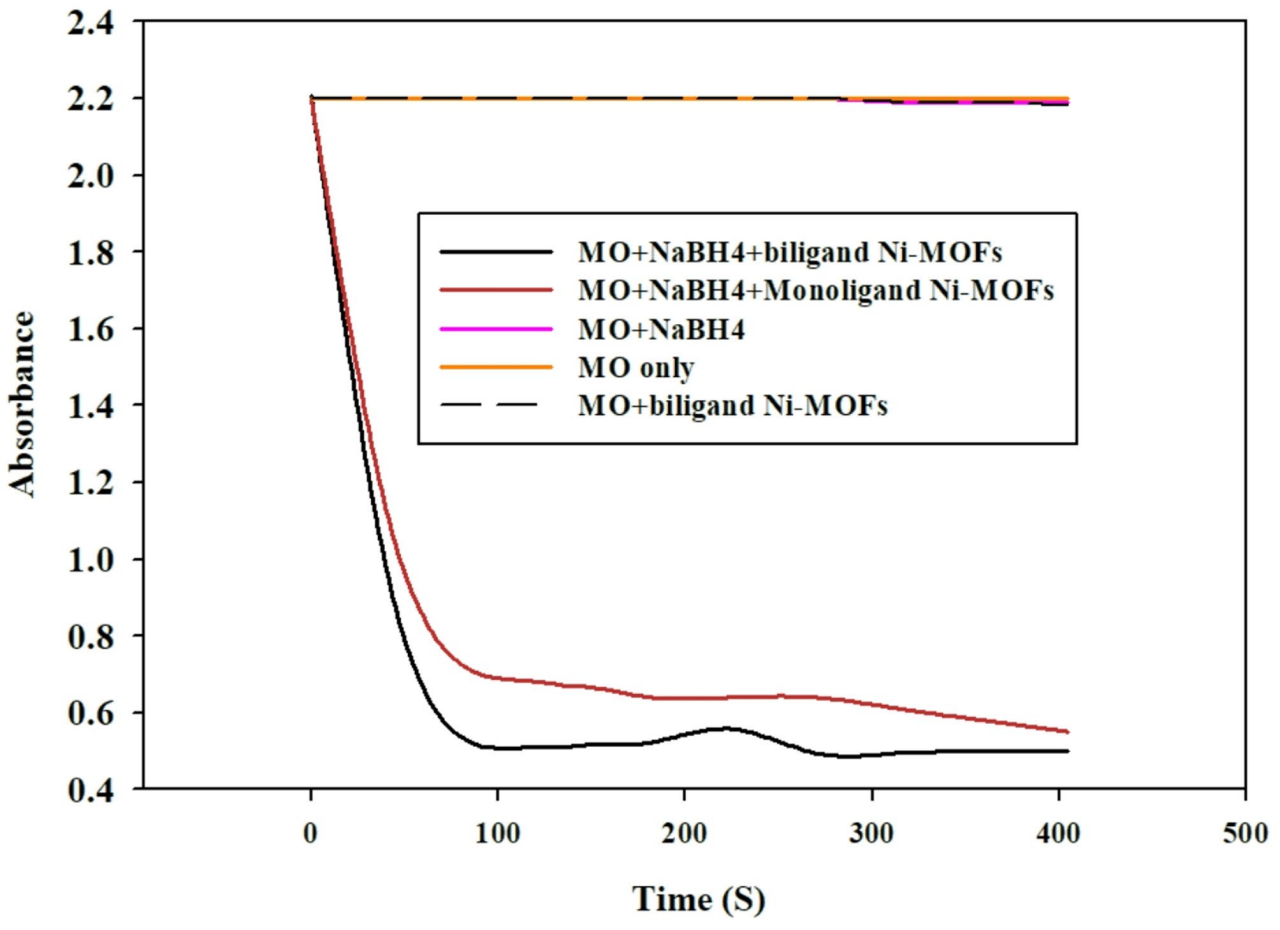
Degradation of MO azodye using NaBH4, with and without the synthesized catalysts monoligand Ni-MOFs, biligand Ni-MOFs for 400 S

The catalytic reduction of MO was investigated using the bi-ligand Ni-MOFs as a nanocatalyst and NaBH₄ as a reducing agent. The findings showed that when using only NaBH₄, the breakdown of MO was slow. This is because a high amount of energy is needed in order to break the stable azo bond (-N = N-) and there aren’t enough active sites where an effective reaction would take place, which depends on random collisions between NaBH₄ and MO, leading to inefficient electron transfer [53]. Similarly, when using only bi-ligand Ni-MOFs, the process was also slow. Even though these Ni-MOFs have MO binding sites, they are unable to provide the necessary hydride ions (H⁻) to break the azo bond (-N = N-). But when bi-ligand Ni-MOFs combined with NaBH₄, the reduction efficiency was significantly improved, and MO degraded faster than if we were to use either of them individually. This shows how important bi-ligand Ni-MOFs are in speeding up the reduction process by providing active sites that improve the interaction between NaBH₄ and MO, boosting the reaction rate. Figure 5 illustrates the improved degradation kinetics observed when the bi-ligand Ni-MOFs are employed in conjunction with NaBH₄.

**Fig. 6:**
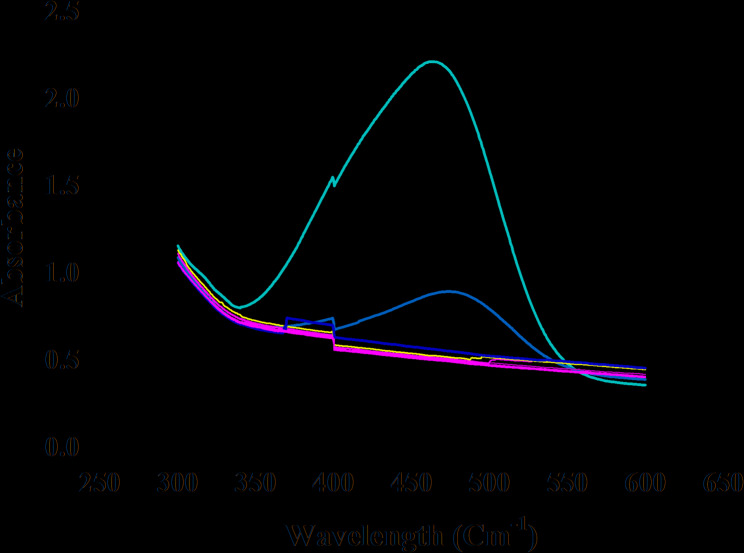
UV-Vis spectra for degradation of MO at 25 °C in presence of NaBH_4_ and bi-ligand Ni-MOFs

Figure 6 presents the absorbance changes over time during the degradation of MO in the presence of bi-ligand Ni-MOFs as a nanocatalyst. It clearly demonstrates the catalytic activity of the bi-ligand Ni-MOFs, as evidenced by the significant decrease in absorbance at the characteristic wavelength of MO (465 nm), which corresponds to the azo group (-N = N-) in the MO dye molecule.

**Fig. 7:**
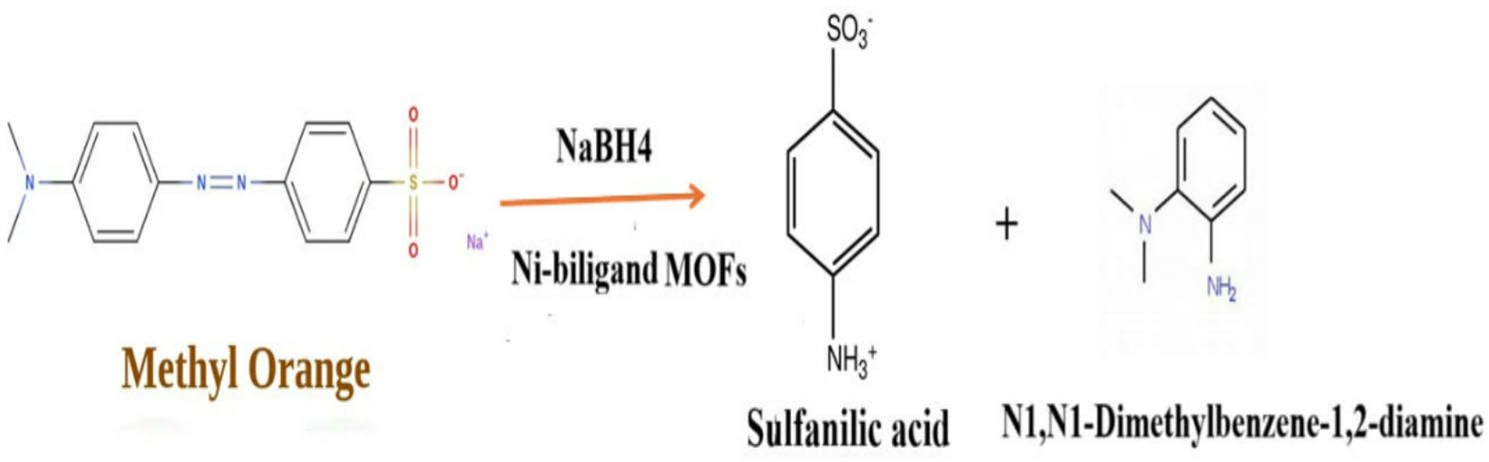
Schematic diagram of MO degradation by NaBH_4_ in presence of bi-ligand Ni-MOFs as nanocatalyst

The proposed reduction mechanism is depicted in Fig. 7. Upon reduction, the azo group (-N = N-) in MO is cleaved, leading to the breakdown of the dye into smaller, colorless products sulfanilic acid and N, N-dimethylbenzene-1,2-diamine. Both are non-toxic and environmentally benign, eliminating the toxic, carcinogenic properties of the original MO molecule. The yellow color of MO was entirely removed within 90 s, confirming the complete reduction of the dye. To establish a baseline, the reduction of MO using NaBH₄ was also examined without adding Ni-MOFs as shown in Fig. 5. In the reduction process, the Ni²⁺ centers in the bi-ligand Ni-MOF play a dual role: they provide coordination sites for the adsorption of MO molecules and simultaneously facilitate the activation of NaBH₄. The nickel sites enhance the transfer of hydride ions (H⁻) from NaBH₄ to the azo bond (–N = N–), thereby accelerating its cleavage. The presence of BTC and PYDC ligands around the Ni²⁺ centers generate a more versatile coordination environment, which not only stabilizes the active sites but also promotes faster electron transfer between the reducing agent and the dye molecules. This synergistic effect of the metal centers and dual ligands results in the observed rapid and efficient degradation of MO, surpassing the performance of mono-ligand systems.

#### Bi-ligand versus mono-ligand Ni-MOFs and previously reported methods

**Fig. 8:**
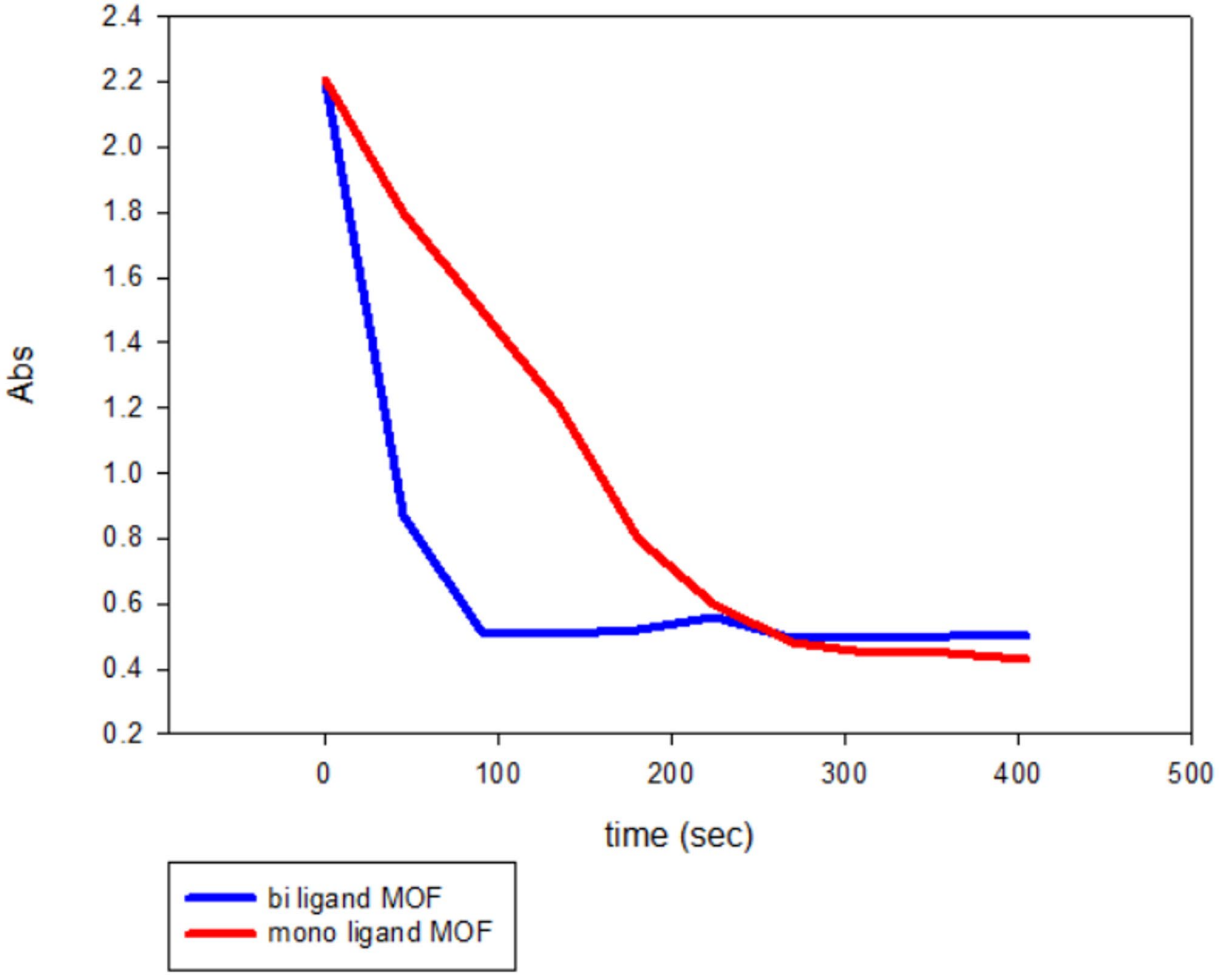
Comparison between bi-ligand and mono ligand Ni-MOFs in degrading MO using NaBH₄ as reducing agent

A comparison between mono- and bi-ligand Ni-MOFs, as illustrated in Fig. 8, proves the superior catalytic performance of the bi-ligand Ni-MOFs, confirming the initial hypothesis that bi-ligand systems would exhibit enhanced catalytic activity compared to their mono-ligand counterparts. This superior performance is attributed to their structural advantages, which include a higher density of active sites and more effective ligand-metal interactions. The incorporation of two distinct ligands in the bi-ligand framework likely creates a more complex and versatile coordination environment, facilitating faster electron transfer and stronger adsorption of reactants. In particular, PYDC plays a crucial role, as its nitrogen atom and dual carboxyl groups provide multiple coordination sites for Ni²⁺, enhancing metal binding, framework stability, and catalytic activity compared to BTC alone [54].

Furthermore, a comparison of the catalytic efficiency of the proposed bi-ligand Ni-MOFs with other reported catalysts for MO reduction, reveals superior performance and significantly faster reaction rates, as summarized in Table 1, although variations in reaction conditions such as NaBH₄ concentration, catalyst loading, and solution volume should be considered when interpreting these results.

**Table 1:** Comparison of the catalytic efficiency of the fabricated bi-ligand Ni-MOFs with previously reported catalysts for reduction of Methyl orange

Catalyst	NaBH4 amount	Catalyst amount	K (S^− 1^)	Reaction Completion Time(min)	Reference
Cu NPs	0.5 mL (0.2 M)	5000 mg	8.6 × 10^− 3^	4	[29]
Pd NPs	1 mL (0.01 M)	4500 mg	1.36–2.2 × 10^− 3^	12–18	[30]
Ag nanostructure	25 mL (0.033 M)	1 cm²	0.56 × 10^− 3^	60	[31]
Ag NPs	0.4 mL (0.1 M)	25–45 mg	14.4 × 10^− 3^	6	[32]
SDS/Ag NPs	0.5 mL (0.025 M)	448 mg mL⁻¹	6.4 × 10^− 3^	21	[33]
CuAg/ZnO/carbon black-cellulose acetate sheets	0.5 mL (1 M)	0.5 cm²	1.5 × 10^− 3^	12	[34]
Poly aniline/NiO composite	N/A	10,000 mg	1.0 × 10^− 3^	30	[35]
MFe_2_O_4_/γ-Fe_2_O_3_ nanocomposites (M = Ni or Co)	0.5 mL (0.3 M)	100 mg	Ni = 1.35 × 10^− 3^ Co = 1.17 × 10^− 3^	36	[36]
C@Fe	1 mL (0.5 M)	5000 mg	15.3 × 10^− 3^	4	[37]
Prussian blue analogue	0.2 mL (0.3 M)	10 µg	38.6 × 10^− 3^	1.75	[2]
Bi-ligand Ni- MOF	0.2 mL (0.3 M)	0.2 mg	17.3 × 10^− 3^	1.5	This work

#### Effect of pH on bi-ligand Ni-MOFs catalytic activity

The impact of pH on the catalytic degradation of MO using a bi-ligand Ni-MOFs nanocatalyst in conjunction with NaBH₄ as a reducing agent was examined at varying pH levels of 5, 7, and 9 to assess the efficiency of MO degradation under varying acidic, neutral and alkaline conditions. The results indicated that the degradation process was most effective at pH 5, which could be attributed to two factors. Firstly, under acidic conditions, NaBH₄ undergoes accelerated hydrolysis, producing hydrogen gas and active hydride ions, which are potent reducing agents facilitating the breakdown of MO molecules. Secondly, the increased concentration of protons at lower pH levels enhances the electron transfer processes between NaBH₄, the nanocatalyst, and MO, thereby accelerating the reduction reaction. ​These findings are relevant as in many industries such as textiles and paper manufacturing, dyeing processes often operate under acidic conditions to ensure proper dye fixation [55, 56]. Consequently, effluents from these processes are acidic. Therefore, the application of this catalytic system under such conditions aligns well with the inherent characteristics of industrial effluents, facilitating more efficient and effective degradation of MO.

#### Reaction order

In our experiment, the bi-ligand Ni-MOFs nanocatalyst employed serves to lower the activation energy and increase the reaction rate without being consumed in the process. Consequently, it does not appear in the rate law and does not influence the reaction order [57]. Similarly, NaBH₄ is utilized in large excess, ensuring its concentration remains effectively constant throughout the reaction. This allows us to treat the reaction as pseudo–first order where the rate depends solely on the concentration of the limiting reactant (MO).

**Fig. 9:**
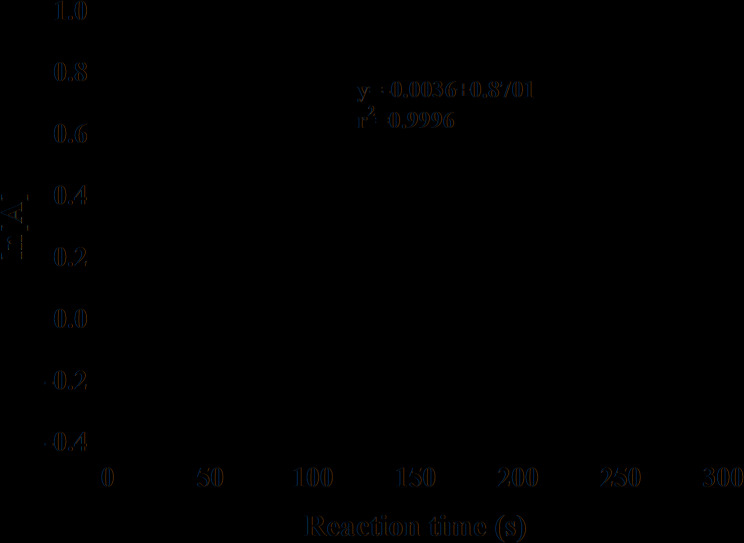
Kinetics plot of ln [A] against reaction time for catalytic reduction of MO using bi-ligand Ni-MOFs

Building upon our reaction order analysis, we conducted spectrophotometric measurements to monitor the catalytic degradation of MO. Absorbance data were recorded over the wavelength range of 300.0–600.0 nm. The results demonstrated that the degradation process adheres to first-order kinetics, as evidenced by the linear relationship with r^2^ = 0.9996 (observed when plotting ln [A] versus time (Fig. 9). This linearity confirms that the reaction rate is directly proportional to the concentration of MO, validating our assumption of pseudo–first-order kinetics under the experimental conditions employed.

### Investigation of Ni-MOFs reusability in the catalytic reduction of MO

**Fig. 10:**
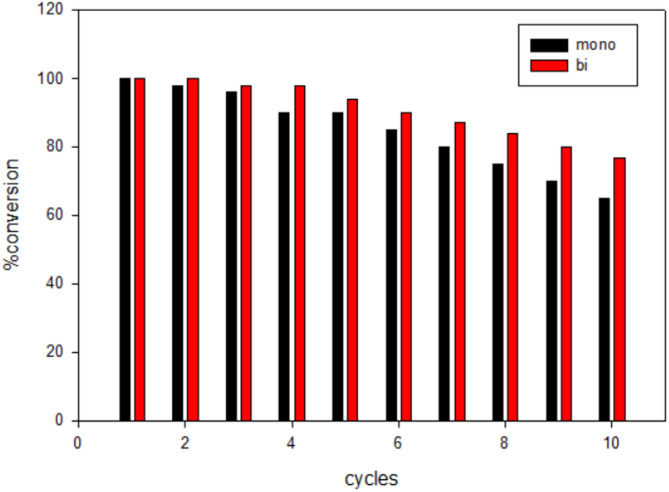
The percentage degradation of MO using mono and bi-ligand Ni-MOFs as nanocatalysts over ten cycles

The reusability of the bi-ligand Ni-MOFs was evaluated and compared to the mono-ligand Ni-MOFs in degrading the dye over multiple cycles, as shown in Fig. 10. The results showed the differences in performance between the two catalysts. The bi-ligand Ni-MOFs kept their performance steady across multiple cycles. In the first round, it took 90 s to degrade the dye, and that time stayed pretty much the same in later rounds. Even after many uses, the bi-ligand catalyst still worked faster than the mono-ligand one. This demonstrates the superior reusability and structural stability of the bi-ligand catalyst, making it a good option for long-term applications.

## Conclusion

The findings of this work demonstrate that integrating two different ligands into the Ni-MOF framework significantly influences the material’s catalytic behavior. The incorporation of both BTC and PYDC ligands not only enhanced nickel loading and active site uniformity but also improved structural stability and surface characteristics, enabling rapid and efficient degradation of methyl orange. The superior performance of the bi-ligand system over the mono-ligand counterpart highlights the importance of ligand selection and coordination chemistry in tailoring MOF properties for specific applications. Beyond dye degradation, the structural and catalytic features observed here suggest that such bi-ligand MOFs could be adapted for other environmental and industrial processes that require fast and reusable catalysts. Future studies may focus on scaling up synthesis, testing under real wastewater conditions, and exploring the versatility of this design strategy for a broader range of pollutants.

## Supplementary Information

Below is the link to the electronic supplementary material.

Supplementary Material 1

## Data Availability

The data will be available upon request.
