# Supplementary material for: Rapid nanocatalytic approach for azo dye degradation using bi-ligand nickle based-metal organic frameworks
Source: BMC Chem. 2025 Oct 27;19(1):287. doi: 10.1186/s13065-025-01650-8 (PMC12560516; doi:10.1186/s13065-025-01650-8)
Supplement: Supplementary file 1 — Supplementary Material 1 [file 13065_2025_1650_MOESM1_ESM.docx]

**Supplementary Material for**

**“Rapid Nanocatalytic Approach for Azo Dye Degradation Using Bi-Ligand Nickle Based-Metal Organic Frameworks”**

Aya A. Mouhamed ^a*^, Ahmed Elsayed ^b^, Noha Mostafa ^b^, Amr M. Mahmoud ^a,b^, Amr Elshaer^c^, Aya T. Soudi ^a^

*^a^* Pharmaceutical Analytical Chemistry Department, Faculty of Pharmacy, Cairo University, El-Kasr El-Aini Street, 11562 Cairo, Egypt.

*^b^* Department of Chemistry, School of Pharmacy, Newgiza University, Egypt

^c^ Drug Discovery, Delivery and Patient Care (DDDPC), School of Life Sciences, Pharmacy and Chemistry, Kingston University London, Kingston Upon Thames, Surrey, KT1 2EE, UK

***Corresponding author’s email:** [**aya.ahmed@pharma.cu.edu.eg**](mailto:aya.ahmed@pharma.cu.edu.eg)

**Table S1.** Materials and reagents

| **Chemical Name** | **Chemical Formula** | **Grade / Purity (%)** | **Supplier** | **Country** |
| --- | --- | --- | --- | --- |
| Nickel(II) chloride hexahydrate Ni(NO₃)₂·6H₂O | NiCl₂·6H₂O | ≥ 98% | Sigma-Aldrich | Germany |
| 1,3,5-Benzenetricarboxylic acid (BTC) | C₉H₆O₆ | ≥ 98% | Sigma-Aldrich | Germany |
| Pyridine-2,3-dicarboxylic acid (PYDC) | C₇H₅NO₄ | ≥ 98% | Sigma-Aldrich | Germany |
| Boric acid | H₃BO₃ | ≥ 99% | Sigma-Aldrich | Germany |
| Phosphoric acid | H₃PO₄ | ≥ 85% | Sigma-Aldrich | Germany |
| Acetic acid | CH₃COOH | ≥ 99.7% | Sigma-Aldrich | Germany |
| Sodium hydroxide | NaOH | ≥ 98% | Sigma-Aldrich | Germany |
| Methyl orange | C₁₄H₁₄N₃NaO₃S | Analytical grade | Sigma-Aldrich | Germany |
| Ethanol (HPLC grade) | C₂H₅OH | ≥ 99.8% | Fisher Scientific | UK |
| Deionized water (DI) | H_2_O | -- | SEDICO Pharmaceuticals Co. | Egypt |

**Figure S1.** HRTEM image of A) biligand Ni-MOFs B) monoligand Ni-MOFs.

**Figure S2.** EDS Mapping for Biligand Ni-MOFs

**Figure S3.** EDS Mapping for Monoligand Ni-MOFs.

**Figure S4.** a) BET surface area measurement and b) Pore size distribution calculated from the desorption branch of the samples employing the NLDFT method of Biligand Ni-MOFs.

**Figure S5.** a) BET surface area measurement and b) Pore size distribution calculated from the desorption branch of the samples employing the NLDFT method of monoligand Ni-MOFs.

**Figure S6.** XRD pattern of A ) biligand Ni-MOFs and B) Mono ligand Ni MOFs
